# A Phase 1 study of the novel gamma-secretase inhibitor PF-03084014 in patients with T-cell acute lymphoblastic leukemia and T-cell lymphoblastic lymphoma

**DOI:** 10.1038/bcj.2015.80

**Published:** 2015-09-25

**Authors:** C Papayannidis, D J DeAngelo, W Stock, B Huang, M N Shaik, R Cesari, X Zheng, J M Reynolds, P A English, M Ozeck, J C Aster, F Kuo, D Huang, P D Lira, K R McLachlan, K A Kern, G Garcia-Manero, G Martinelli

**Affiliations:** 1 Department of Experimental, Diagnostic, and Specialty Medicine, University of Bologna, Bologna, Italy; 2 Adult Leukemia Institute, Dana-Farber Cancer Institute, Boston, MA, USA; 3 Department of Medicine, Hematology/Oncology Section, University of Chicago, Chicago, IL, USA; 4 Pfizer Oncology, Groton, CT, USA; 5 Pfizer Oncology, San Diego, CA, USA; 6 Pfizer Oncology, Milan, Italy; 7 Department of Pathology, Dana Farber Cancer Institute, Boston, MA, USA; 8 Department of Leukemia, The University of Texas MD Anderson Cancer Center, Houston, TX, USA

T-cell acute lymphoblastic leukemia (T-ALL) and T-cell lymphoblastic lymphoma (T-LBL) are aggressive malignancies derived from neoplastic transformation of precursor T cells. T-ALL accounts for 10–15% of pediatric and 25% of adult ALL cases.^1^ Although current treatment with intensive chemotherapy regimens may achieve a cure rate of 80% in children with T-ALL, treatment of adult patients leads to a lower response rate.^2^

Results from a number of studies have demonstrated a key role for the deregulation of NOTCH signaling pathways in the oncogenic transformation leading to the development of T-ALL and T-LBL, providing a rationale for the development of gamma-secretase inhibitors as a novel targeted therapy for these hematologic malignancies.^3, 4, 5, 6, 7^ PF-03084014 is a noncompetitive, reversible, targeted agent that selectively inhibits gamma secretase in multiple tumor types including leukemia and lymphoma.^8, 9, 10^ Treatment with PF-03084014 has demonstrated significant antitumor activity in preclinical models of lymphoid malignancies and solid tumors.^8, 9, 10^

In study A8641014 (approved by the ethics and regulatory committees of the participating institutions, with signed patient informed consent, and following the Declaration of Helsinki and good clinical practice guidelines), eight patients with T-ALL or T-LBL received treatment with PF-03084014 150 mg twice daily in continuous cycles, and all were evaluable for safety and treatment response as well as pharmacokinetic and pharmacodynamic analyses. Mean patient age was 31 (range 18–43) years. Six patients were male and two were female; the majority (75%) was white (Table 1). Five patients had a primary diagnosis of T-LBL with a mean duration of 2.4 years and three had a primary diagnosis of T-ALL with a mean duration of 4.8 years. All eight patients had received prior systemic therapy; most patients (*n*=7, 87.5%) had been treated with two or three prior regimens and three (37.5%) had received prior radiation therapy.

Treatment with PF-03084014 was associated with a complete response (CR) in a patient with T-ALL that lasted for ~3 months, with evidence of full hematologic recovery. This patient had been heavily pretreated, achieving a CR following induction therapy with cyclophosphamide, idarubicin, methotrexate, vincristine and dexamethasone, but not after salvage treatment with nelarabine, vincristine and cyclophosphamide; the patient also relapsed after a cord blood stem cell transplant performed 8 months before study entry.

*NOTCH1* sequence analysis by conventional Sanger sequencing did not reveal any *NOTCH1* mutation in the peripheral blood samples from five patients, including the T-ALL patient with a CR. Evaluation by the more sensitive deep-sequencing method revealed a known activating mutation, L1679P, in exon 27 of *NOTCH1* in the T-ALL patient with a CR, which was confirmed in an independent bone marrow sample collected at a different time point (25% blasts; Table 2). This finding is consistent with the hypothesis that *NOTCH1*-activating mutations may have a leukemogenic role in T-ALL and confer sensitivity to gamma-secretase inhibition. However, deep-sequencing analysis also revealed a known activating *NOTCH1* mutation (V1677D) in bone marrow mononuclear cells from a non-responding patient with T-LBL. This suggests that mutation status does not consistently predict response to PF-03084014, in line with prior clinical trials.^3^ Further, it may indicate differences in the biology of the disease and in the role played by NOTCH-mediated signaling pathways in T-LBL versus T-ALL (for example, the degree of 'NOTCH addiction' in tumor cells) or, alternatively, a resistance to treatment with gamma-secretase inhibitors in T-LBL cells, mediated by other pathways (for example, PI3/mTOR kinase signaling).^11, 12^

The most common adverse events following treatment with PF-03084014 in patients with T-ALL/T-LBL were nausea and vomiting. Diarrhea was not a treatment-limiting toxicity in patients with T-ALL/T-LBL, as previously observed with other investigational gamma-secretase inhibitors^13, 14^ and it was mostly low grade (grades 1 and 2). In contrast with the results obtained in patients with solid tumors,^15^ no rash and hypophosphatemia were reported in patients with T-ALL/T-LBL treated with PF-03084014, although the duration of treatment was shorter and the number of treated patients was substantially lower in this population. The causality of the dose limiting toxicity reported in this study, elevations in liver enzymes, remains unclear as it was observed in a patient who was receiving concomitant treatment with hepatotoxic drugs, and had chronic graft-versus-host disease and a suspected hepatic infection (candidiasis). Furthermore, no hepatic enzyme abnormalities (AST and ALT) and bilirubin elevations were noted when PF-03084014 administration was restarted at the reduced dose of 130 mg twice daily and continued for at least 2 months.

Pharmacokinetic analysis of PF-03084014 following single-dose and multiple-dose administration to patients with T-ALL or T-LBL demonstrated a favorable pharmacokinetic profile. Steady state was achieved by day 8 of treatment and the mean terminal half-life was 18 h (s.d., 3.6), after repeated daily dosing of PF-03084014.

Treatment with PF-03084014 induced inhibition of *HES4* gene expression levels at days 8, 15 and 21 of cycle 1 in the peripheral blood (as surrogate tumor tissue with no leukemic blast separation) of the majority of patients with T-ALL and T-LBL, thus providing a biomarker for measuring *in vivo* modulation of NOTCH pathway-related targets. Of note, *HES4* gene expression levels were inhibited throughout cycle 1 in the responding patient with T-ALL, with an increase above baseline levels at disease relapse (end-of-treatment sample).

In conclusion, the anti-T-ALL activity demonstrated by PF-03084014 in this study, as well as the antitumor activity observed in patients with solid tumors,^15^ supports further evaluation of PF-03084014 in patients with T-ALL or T-LBL in an earlier therapeutic setting to limit the confounding factor represented by the poor prognosis associated with relapsed or refractory disease.

## Figures and Tables

**Table 1:** Patient baseline demographics and clinical characteristics

*Parameter*	*Patients (*N=*8)*
Age, years, mean (range)	30.8 (18–43)
Male:female	6:2
	
*Race*, n *(%)*	
White	6 (75.0)
Black	1 (12.5)
Asian	0
Other	1 (12.5)
	
*ECOG PS*, n *(%)*	
0	3 (37.5)
1	4 (50.0)
2 (at baseline), 1 (at screening)	1 (12.5)
	
*Primary diagnosis*, n *(%)*	
T-LBL	5 (62.5)
T-ALL	3 (37.5)
	
*Disease duration since diagnosis, years, mean (range)*	
T-LBL	2.4 (0.4–7.4)
T-ALL	4.8 (0.6–12.3)
	
*Prior radiation therapy*, n *(%)*	
Yes	3 (37.5)
No	5 (62.5)
	
*Prior systemic therapies*, n *(%)*	
Yes	8 (100)
	
*Systemic regimens*, n *(%)*	
1	1 (12.5)
2	3 (37.5)
3	4 (50.0)

Abbreviations: ECOG PS, Eastern Cooperative Oncology performance status; T-ALL, T-cell lymphoblastic leukemia; T-LBL, T-cell lymphoblastic lymphoma.

**Table 2:** *NOTCH1* mutations detected by deep sequencing in PF-03084014-treated patients

*Primary diagnosis*	*Sample type*	*Blast (%)*	*Sequencing method*	*NOTCH1 mutation detected*	*Domain*
T-ALL	Bone marrow	75	Ion torrent	None detected	Not applicable
T-LBL	Bone marrow	11	Ion torrent	V1677D	HD
		11	Ion torrent	V2444fs*35	PEST
T-ALL	Bone marrow	Undetectable	Ion torrent	L1679P	HD
		25	Ion torrent	L1679P	HD
T-LBL	Bone marrow	Undetectable	Ion torrent	V1672I	HD (SNP)

Abbreviations: HD, heterodimerization domain; PEST, proline-, glutamic acid-, serine-, and threonine-rich domain; SNP, single-nucleotide polymorphism; T-ALL, T-cell lymphoblastic leukemia; T-LBL, T-cell lymphoblastic lymphoma.
